# Understanding nerve–tumor interactions: From basic biology to therapeutic innovation

**DOI:** 10.1016/j.gendis.2025.101955

**Published:** 2025-11-28

**Authors:** Liangzhan Sun, Xia Li, Yaxuan Wang, Jingxuan Wang, Renrui Xie, Ningyi Zhang, Zemin Zhang

**Affiliations:** a Peking University Shenzhen Graduate School, Peking University, Shenzhen, Guangdong 518055, China; b Institute for Data-Driven Tumor Immunology, Chongqing Medical University, Chongqing 400016, China; c School of Basic Medical Sciences, Chongqing Medical University, Chongqing 400016, China; d Department of Molecular Microbiology and Immunology, Johns Hopkins Bloomberg School of Public Health, Johns Hopkins University, Baltimore, MD 21205, USA; e School of Medicine, The Chinese University of Hong Kong-Shenzhen, Shenzhen, Guangdong 518117, China

**Keywords:** Cancer cell, Immune cell, Nerve, Tumor, Tumor microenvironment

## Abstract

The study of nerve–tumor interactions has emerged as a rapidly advancing and interdisciplinary field with profound implications for understanding cancer progression, prognosis, and therapeutic innovation. While this area holds significant promise for transformative discoveries, the mechanisms of nerve–tumor interactions and their translation into clinical applications remain at an early stage. This review focuses on the role of peripheral nerves in non-neurogenic solid tumors, discussing the prevalence and clinical impact of nerve–tumor interactions, their underlying forms and mechanisms, advancements in research technologies, therapeutic potential, and future challenges. By synthesizing current knowledge, integrating methodologies for studying nerve–tumor interactions, and identifying critical gaps, this work aims to provide a foundational resource to guide experimental design and stimulate interest in clinical trials targeting neural influences in cancer progression.

## Introduction

**Figure 1:**
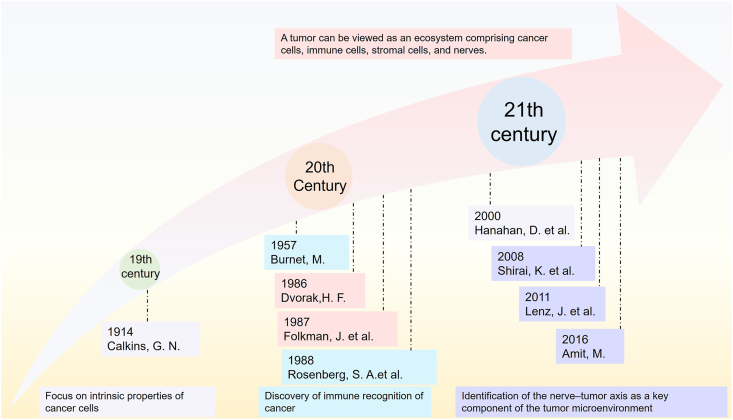
History and milestones of cancer research.

The understanding of cancer has evolved significantly over the past century, shifting from a narrow focus on intrinsic cellular mechanisms to a broader appreciation of the tumor as a complex ecosystem^1^, ^2^, ^3^, ^4^, ^5^, ^6^, ^7^, ^8^ (Fig. 1). In the late 19th and early 20th centuries, cancer was predominantly viewed as a disease caused by uncontrolled cell proliferation, driven by mutations in key regulatory genes.^2^^,^^3^ Research during this period concentrated on elucidating the intrinsic properties of cancer cells, laying the foundation for modern oncology.

As the understanding of cancer biology deepened, attention gradually shifted beyond cancer cells themselves to the role of the host immune system. The concept of immune surveillance, proposed in 1957, introduced the idea that the immune system could recognize and eliminate cancer cells.^1^ However, it was not until the late 20th century that the intricate relationship between tumors and the immune system was widely recognized.^4^ This led to the development of the tumor immune microenvironment concept, highlighting the dynamic interplay between tumor cells and immune components. The growing understanding of this interplay catalyzed the development of immunotherapies, including checkpoint inhibitors such as anti-PD-1 and anti-CTLA-4, which revolutionized cancer treatment in the 2010s.^7^^,^^8^

In parallel, the role of stromal cells, including fibroblasts, endothelial cells, and adipocytes, and the extracellular matrix (ECM) gained prominence during the 1980s and 1990s.^5^^,^^6^ These discoveries underscored the importance of stromal components in supporting tumor growth, angiogenesis, and metastasis, reframing the tumor as an ecosystem comprising cancer cells, immune cells, and the stroma.^5^^,^^6^ This understanding facilitated the development of stroma-targeting therapies, such as anti-angiogenic drugs like bevacizumab, which inhibits vascular endothelial growth factor (VEGF).^9^

More recently, studies from the late 2010s have identified the nerve–tumor axis as a critical component of the tumor microenvironment (TME).^10^, ^11^, ^12^ Nerve-derived signals have been implicated in promoting cancer cell proliferation, modulating the immune microenvironment, driving metastasis, and contributing to therapy resistance.^13^, ^14^, ^15^, ^16^ These findings have opened new avenues for therapeutic intervention, including targeting neural signals and nerve-associated growth factors.

**Figure 2:**
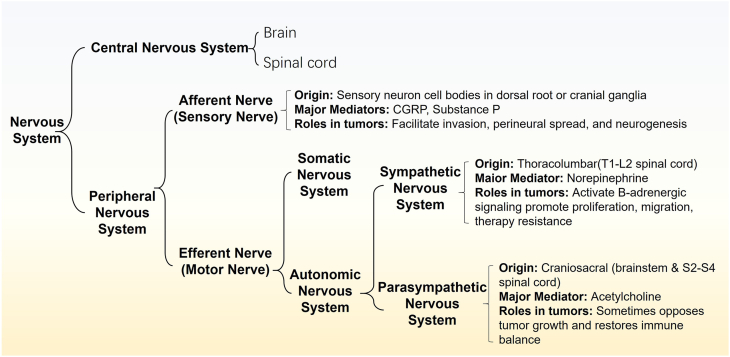
Schematic of nervous system classification.

The nervous system comprises the central nervous system and the peripheral nervous system (Fig. 2). The peripheral system is further subdivided into autonomic and somatic–sensory divisions. The autonomic division includes the sympathetic branch, which operates predominantly through adrenergic signaling; the parasympathetic branch, which relies largely on cholinergic signaling; and the enteric branch, which regulates gastrointestinal function and broader visceral homeostasis.^17^ The somatic–sensory division comprises afferent sensory fibers, including nociceptors that detect noxious and inflammatory stimuli, and somatic motor fibers that innervate skeletal muscle.^18^ In this review, we focus on peripheral autonomic fibers (sympathetic, parasympathetic, and enteric) and sensory afferents that innervate non-neurogenic solid tumors, as these inputs most directly modulate tumor cells as well as the immune and stromal compartments of the TME. This review provides an in-depth analysis of nerve–tumor interactions in non-neurogenic solid tumors, with a particular focus on their prevalence, clinical relevance, and the underlying molecular mechanisms. Additionally, it discusses emerging technologies, therapeutic potentials, and the challenges that lie ahead in this rapidly evolving field. By synthesizing current knowledge, the review aims to foster further research and contribute to the development of novel therapeutic strategies targeting the neural microenvironment in cancer. It is worth noting that Schwann cells, which are responsible for the myelination of peripheral nerves, are not included in this review, as their involvement in tumor biology has been comprehensively addressed in several high-quality, dedicated reviews.^19^, ^20^, ^21^

## Types of nerve–tumor interactions

**Figure 3:**
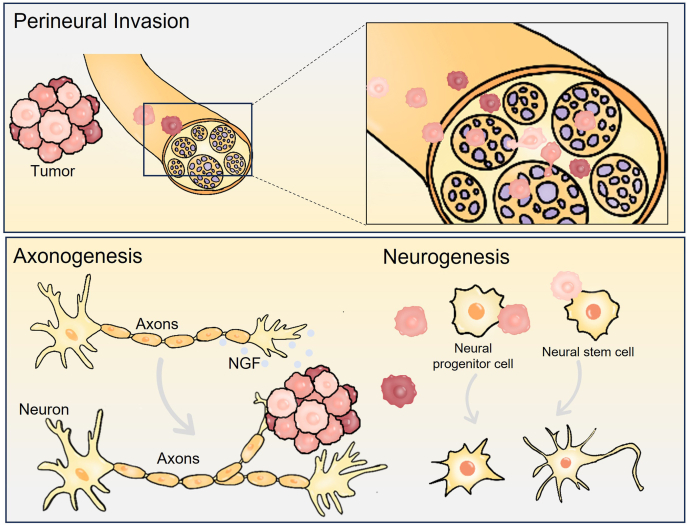
Types of nerve–tumor interactions.

Nerve–tumor interactions represent a critical aspect of cancer progression. These interactions manifest through diverse mechanisms, such as perineural invasion (PNI), axonogenesis, and neurogenesis, which collectively contribute to tumor growth, local invasion, and distant metastasis by fostering a dynamic crosstalk between the nervous system and tumor cells (Fig. 3).^22^, ^23^, ^24^

## Perineural invasion

PNI is a pathological process where cancer cells invade and spread along nerves. As one of the most extensively studied forms of nerve–tumor interaction, PNI is a prevalent feature of nerve–tumor interactions, observed in 68.8%–100% of pancreatic cancer cases,^11^^,^^25^, ^26^, ^27^, ^28^ 20.7%–81.4% of cholangiocarcinoma cases,^12^^,^^29^, ^30^, ^31^, ^32^, ^33^ 31.7%–75.6% of gastric cancer cases,^34^, ^35^, ^36^, ^37^, ^38^ and 7.0%–75% of prostate cancer cases.^39^, ^40^, ^41^, ^42^, ^43^, ^44^, ^45^, ^46^, ^47^, ^48^, ^49^, ^50^, ^51^, ^52^, ^53^, ^54^ These high incidence rates indicate that PNI represents the most common manifestation of nerve involvement across these malignancies (Table 1). Importantly, beyond its frequency, PNI also bears considerable prognostic value. It has significant clinical implications across multiple cancers, including pancreatic cancer,^55^ oral squamous cell carcinoma,^56^ head and neck squamous cell carcinoma,^57^ breast cancer,^58^ laryngeal cancer,^59^ rectal cancer,^60^ gastric cancer,^61^^,^^62^ and colorectal cancer,^63^ where its presence has been associated with poorer patient survival (Table 2). Moreover, the presence of PNI has been strongly linked to metastatic potential in several malignancies, including oral squamous cell carcinoma,^56^ head and neck squamous cell carcinoma,^57^ breast cancer,^58^ rectal cancer,^60^ gastric cancer,^61^^,^^62^ and colorectal cancer.^63^ In particular, in oral squamous cell carcinoma,^56^ head and neck squamous cell carcinoma,^57^ and gastric cancer,^61^^,^^62^ PNI is not only predictive of metastasis but is also correlated with advanced pathological staging, underscoring its role in tumor progression and disease severity (Table 2). Beyond prognostic associations, accumulating evidence indicates that PNI contributes meaningfully to cancer-related pain across multiple tumors, particularly pancreatic ductal adenocarcinoma and head-and-neck/oral squamous cell carcinomas, with supportive signals also emerging in colorectal cancer (Table 2).^64^, ^65^, ^66^, ^67^ Evidence from multiple studies indicates that higher PNI burden associates with greater pain intensity, and in some contexts, pretreatment pain predicts PNI presence or severity.^68^

**Table 1:** The incidence of different forms of nerve–tumor interactions.

Cancer type	Incidence rate	Reference
*Perineural invasion*		
Pancreatic cancer	68.8%–100%	11,25, 26, 27, 28
Cholangiocarcinoma	81.4%	12,29, 30, 31, 32, 33
Gastric cancer	31.7%–75.6%	34, 35, 36, 37, 38
Prostate cancer	7.0%–75%	39, 40, 41, 42, 43, 44, 45, 46, 47, 48, 49, 50, 51, 52, 53, 54
Parotid gland malignancies	46%	128
Cervical cancer	8.6%–35.1%	271, 272, 273
Squamous cell carcinoma of the larynx and hypopharynx	33.6%	274
Oral tongue cancer	20.3%–30%	275, 276, 277, 278
Esophagus carcinoma	29%	129
Breast carcinoma	1.14%–25.7%	71,279
Bladder cancer	24.4%	280, 281, 282
Rectal cancer	17.1%–24.3%	130,283, 284, 285, 286
Colorectal cancer	8.9%–22.6%	63,127,287, 288, 289, 290, 291, 292
Oropharyngeal carcinoma	15.9%	293
Non-small-cell lung cancer	9.0%	294
Transitional cell carcinoma of the bladder	8.8%	295
Cutaneous squamous cell carcinoma	5.96%	72
Basal cell carcinoma	2.2%–2.74%	296,297
*Axonogenesis*		
Pancreatic ductal adenocarcinoma	71.1%	78
Prostate cancer	68%	79
*Neurogenesis*		
Colorectal cancer	63%	83
Breast cancer	61.8%	84

**Table 2:** The clinical significance of different forms of nerve–tumor interactions.

Interaction type	Cancer type	Prognosis					Reference
		Survival	Recurrence	Metastasis	Clinical stage	Pain	
Perineural invasion	Oral squamous cell carcinoma	Yes (OS & DFS)	Yes	Yes	Yes	Yes	56,66
	Head and neck squamous cell carcinoma	Yes (OS)		Yes	Yes	Yes	57,65
	Breast cancer	Yes (OS & DFS)	Yes	Yes			58
	Laryngeal cancer	Yes (OS & DFS)	Yes				59
	Rectal cancer	Yes (OS)	Yes	Yes			60
	Gastric cancer	Yes (OS & DFS)	Yes	Yes	Yes		61,62
	Squamous cell carcinoma of the thyroid	Yes (OS)					298
	Pancreatic cancer	Yes (OS)	Yes	Yes		Yes	25,55,64
	Hilar cholangiocarcinoma	Yes (OS)					131
	Prostate cancer	Yes (OS & DFS)					44
	Colorectal cancer (stage II)	Yes (OS & DFS)		Yes		Yes	63,67
	Laryngeal cancer	Yes (OS & DFS)					59
Axonogenesis	Prostate cancer	Yes (recurrence-free survival)		Yes			79
Neurogenesis	Pancreatic ductal adenocarcinoma	Yes (OS)	Yes				82
	Colorectal cancer	Yes (OS & DFS)					83
	Invasive ductal carcinoma	Yes		Yes	Yes		84

Note: Only patient-based clinical studies are included. Associations are listed only when statistically significant (*P* < 0.05) in univariable or multivariable analyses. Endpoints are reported exactly as defined in the original studies (*e.g.*, recurrence, metastasis, clinical stage, OS/overall survival; DFS/disease-free survival).

Despite its high prevalence and clinical relevance, the characterization and diagnostic assessment of PNI remain areas of ongoing debate. While its presence is commonly associated with aggressive tumor behavior and poor prognosis, there is still no universally accepted definition or diagnostic standard. This lack of consensus contributes to substantial variability in how PNI is identified and reported across studies and clinical institutions.^29^^,^^42^^,^^63^^,^^69^, ^70^, ^71^, ^72^ Different investigators and pathologists adopt varying definitions—some restrict PNI to cases where cancer cells are found within the nerve sheath, while others include instances where tumor cells are merely surrounding the nerve.^29^^,^^42^^,^^63^^,^^69^, ^70^, ^71^, ^72^ Such inconsistencies not only complicate the interpretation of PNI across studies but also hinder the establishment of its true clinical value. To address this, establishing unified diagnostic criteria is essential to enhance consistency, comparability, and reproducibility in both clinical and research settings.

Moreover, technical limitations in tissue sampling further complicate the reliable detection of PNI. Specifically, small biopsies or limited tissue sections may fail to capture the full extent of tumor–nerve interactions, resulting in potential underdiagnosis. Therefore, minimizing underdiagnosis and improving detection strategies, such as using more comprehensive sampling protocols or advanced imaging and histological techniques, will be crucial for better understanding the pathological relevance of PNI and for guiding its integration into clinical decision-making. Moving forward, these efforts are expected to enhance the diagnostic accuracy and therapeutic stratification associated with PNI-positive tumors.

## Axonogenesis

Axonogenesis refers to the formation of new axonal projections from existing neurons, a fundamental process in neural development and regeneration.^73^ In the context of cancer, axonogenesis describes the extension of axons from pre-existing nerves into the TME, a phenomenon increasingly recognized as a critical component of nerve–tumor crosstalk.^74^^,^^75^ Tumors can secrete various neurotrophic factors, including nerve growth factor (NGF) and brain-derived neurotrophic factor (BDNF), along with other molecular cues, which collectively stimulate aberrant axonal sprouting.^75^, ^76^, ^77^ This tumor-induced axonogenesis facilitates reciprocal interactions between nerves and cancer cells, contributing to tumor growth, local invasion, and metastatic dissemination.

Clinically, axonogenesis has been observed in a significant proportion of tumors, occurring in 71.1% of pancreatic ductal adenocarcinoma cases^78^ and 68% of prostate cancer cases.^79^ Notably, in pancreatic cancer, the presence of axonogenesis correlates with poor clinical outcomes and reduced overall survival, positioning it as a potential prognostic marker and therapeutic target.^78^ These data emphasize the pathological relevance of axonogenesis as not only a hallmark of tumor progression but also a contributor to the aggressive behavior of certain malignancies.

Recent advances in imaging technologies, such as high-resolution live imaging and multiphoton microscopy, have significantly enhanced our ability to study axonal dynamics in real time.^80^ In parallel, the development of brain and neural organoid models offers physiologically relevant platforms to interrogate axonogenesis *in vitro*, bridging the gap between traditional cell culture and *in vivo* systems.^81^ Together, these technological innovations have expanded our mechanistic understanding of axonogenesis in both physiological and pathological settings.

However, several translational challenges persist. While preclinical studies have uncovered important aspects of axonogenesis, its integration into clinical oncology remains limited. A more comprehensive understanding of how axonal outgrowth interfaces with cancer cells, stromal components, and immune infiltrates is necessary to elucidate its multifaceted role within the TME. Addressing these gaps will be critical for harnessing axonogenesis as a therapeutic target in oncology and beyond.

## Neurogenesis

Neurogenesis refers to the generation of new neurons from neural progenitor or stem cells, a process essential for normal development and tissue repair. Within the TME, neurogenesis refers to the phenomenon whereby cancer cells stimulate the formation of *de novo* nerves, distinct from axonogenesis, which involves the sprouting of axons from existing neurons. Tumor-induced neurogenesis has been documented across multiple malignancies, including in 68% of prostate cancer patients,^82^ 63% of colorectal cancer patients,^83^ and 61.8% of breast cancer patients.^84^ These high prevalence rates underscore its emerging relevance as a pathological feature of solid tumors. Importantly, neurogenesis is strongly associated with poor clinical outcomes. In the aforementioned cancers, its presence correlates with significantly reduced patient survival.^82^, ^83^, ^84^ Moreover, in breast cancer, the degree of neurogenesis has been shown to increase with tumor grade, progressing from grade I to grades II and III.^84^ Collectively, these findings highlight the potential of neurogenesis to serve as both a prognostic biomarker and a modifiable target for therapeutic intervention.

Despite its clinical potential, the study of tumor-associated neurogenesis remains limited by several unresolved challenges. First, substantial variability in neurogenesis prevalence across cancer types complicates efforts to define its generalizable biological significance. Second, the molecular and cellular mechanisms underlying neurogenesis in the TME remain poorly characterized. Critical questions, such as the cellular origin of newly formed nerves, the recruitment of neuronal progenitors, and the identity of tumor-secreted neurogenic factors, remain unanswered. In addition, as with axonogenesis, current methodologies for detecting and quantifying neurogenesis are labor-intensive, lack standardized criteria, and often rely on static histological analysis, limiting reproducibility and cross-study comparability.

To advance this field, several key areas must be prioritized. Standardizing detection protocols and establishing quantitative metrics for neurogenesis will be essential for validating its role as a clinical biomarker. Expanding research across diverse cancer types may uncover both shared pathways and tumor-specific mechanisms driving neurogenesis, thereby informing tailored therapeutic approaches. Furthermore, the integration of advanced model systems, such as tumor–nerve co-cultures, patient-derived organoids, and intravital imaging, will facilitate functional dissection of neurogenesis and its contribution to tumor growth, metastasis, and therapeutic resistance. These efforts will be instrumental in translating neurogenesis-related discoveries into clinically actionable strategies aimed at improving cancer outcomes.

## Mechanisms of nerve–tumor interactions

Having established the diverse types of nerve–tumor interactions, a deeper understanding of the underlying mechanisms is critical to unravel how neural signals modulate the TME and drive disease progression. This section explores the molecular and cellular pathways through which nerves influence cancer biology.

## Nerve–cancer cell crosstalk

**Figure 4:**
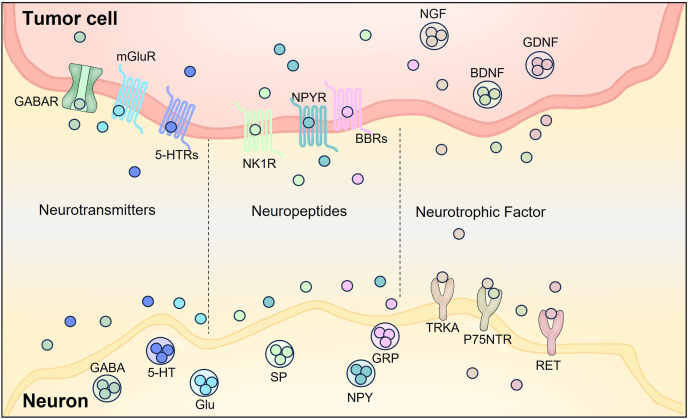
Modes of neural regulation of cancer cells.

**Figure 5:**
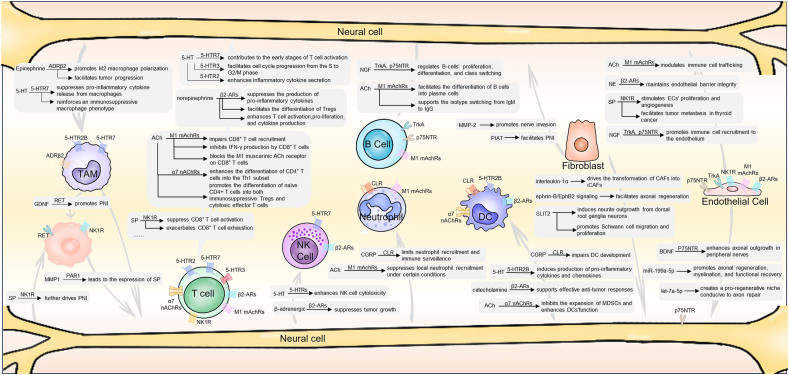
Mechanisms of nerve–tumor interactions.

Cancer cells drive tumor initiation and progression and actively orchestrate the composition and dynamics of the TME. Their capacity for unchecked proliferation, resistance to apoptosis, and adaptability to diverse environmental stressors enable them to sustain tumor growth and facilitate metastasis.^3^ Notably, accumulating evidence suggests that the nervous system can further modulate these malignant behaviors, significantly influencing cancer cell proliferation, metastasis, therapy resistance, and interactions with the TME.^14^^,^^16^^,^^85^^,^^86^ Elucidating the underlying mechanisms of this nerve–cancer cell crosstalk is essential for advancing our understanding of tumor progression and identifying novel therapeutic targets. The following sections explore key pathways through which nerves regulate cancer cell biology and reshape the TME (Figure 4, Figure 5).

### Neural modulation of cancer cells

#### Neurotransmitters

Nerves release various neurotransmitters, such as γ-aminobutyric acid (GABA), norepinephrine, epinephrine, dopamine, serotonin (5-HT), glutamate, histamine, and acetylcholine. These neurotransmitters bind to specific receptors on cancer cells, activating intracellular signaling pathways that regulate tumor progression by influencing cell proliferation, apoptosis, and migration (Table 3).

**Table 3:** Functional effects of neurotransmitter–receptor signaling across cancer types.

Ligand	Receptor	Cancer type	Function	Reference
GABA	GABAA	Gastric cancer	Promotes proliferation	15
		Hepatocellular carcinoma	Inhibits migration	14
	GABRA3	Breast cancer	Promotes migration and invasion	98
	GABRP	Pancreatic ductal adenocarcinoma	Promotes growth	105
	GABAB	Prostate cancer	Promotes metastasis	88
		Hepatocellular carcinoma	Inhibits growth	87
		Colon carcinoma	Inhibits migration	89
		Breast carcinoma	Regulates migration	90,91
		Gastric cancer	Inhibits carcinogenesis	299
Norepinephrine	β2-adrenoceptor	Colon carcinoma	Induces locomotion	101
		Breast cancer	Induces migration	100
	β1- and β2-adrenoceptor	Pancreatic cancer	Promotes invasion	103
Epinephrine	β1- and β2-adrenoceptor	Esophageal squamous cell carcinoma	Stimulates proliferation	300
Dopamine	Dopamine D2 receptor	Breast cancer	Induces migration	100
Serotonin (5-HT)	5-HTR1A	Bladder cancer	Inhibits proliferation	96
		Prostate cancer	Stimulates proliferation	92
		Cholangiocarcinoma	Promotes growth	94
	5-HTR1B	Bladder cancer	Inhibits proliferation	96
	5-HTR2A	Breast cancer	Promotes growth	93
		Cholangiocarcinoma	Promotes growth	94
	5-HTR2B	Prostate cancer	Stimulates proliferation	95
		Cholangiocarcinoma	Promotes growth	94
	5-HTR3A	Colitis-associated colorectal cancer	Promotes progression	102
	5-HTR4	Prostate cancer	Stimulates proliferation	95
		Cholangiocarcinoma	Promotes growth	94
	5-HTR6	Cholangiocarcinoma	Promotes growth	94
	5-HTR7	Breast cancer	Inhibits proliferation	99
Glutamate	GluR1	Breast cancer	Promotes proliferation	97
	GluR2	Glioblastoma multiforme	Inhibits proliferation	301
	GluR3	Pancreatic cancer	Reduces apoptosis; enhances proliferation and migration	104
	GluR4	Colon cancer	Increases 5-fluorouracil resistance	16
	GluR5	Laryngeal cancer	Stimulates proliferation	302
Histamine	H1R	Melanoma	Chemotactic effect	303
Acetylcholine	M3R	Small-cell lung cancer	Increases proliferation	304

Note: GABA, γ-aminobutyric acid.

The effects of neurotransmitters on cancer cells are highly context-dependent, varying with both the type of receptor engaged and the specific tumor type. The same neurotransmitter can elicit distinct, and sometimes opposing, biological outcomes depending on the receptor subtype it binds to. For instance, GABA promotes gastric cancer cell proliferation through activation of GABAA receptors, while signaling through GABAB receptors suppresses cell proliferation in hepatocellular carcinoma.^15^^,^^87^ Similarly, GABAA activation inhibits migration in hepatocellular carcinoma cells, whereas GABAB signaling reduces cell migration in prostate cancer, hepatocellular carcinoma, colon carcinoma, and breast carcinoma. In contrast, activation of the GABRA3 subtype has been shown to enhance migration in breast cancer cells.^14^^,^^88^, ^89^, ^90^, ^91^ A comparable receptor-dependent pattern is observed in 5-HT signaling. Activation of 5-HTR1A promotes proliferation in prostate cancer, while 5-HTR2A facilitates growth in breast cancer and cholangiocarcinoma, and 5-HTR4 contributes to proliferation in prostate cancer.^92^, ^93^, ^94^, ^95^ However, 5-HTR signaling can also exert inhibitory effects; in bladder cancer, 5-HTR1A activation suppresses tumor cell proliferation, and in breast cancer, 5-HTR7 mediates antiproliferative activity.^96^^,^^97^

Within the same tumor type, it is common for multiple receptor subtypes of a given neurotransmitter to be co-expressed. These receptors may exert redundant or synergistic functions. For example, in bladder cancer, both 5-HTR1A and 5-HTR1B inhibit tumor cell proliferation.^96^ In prostate cancer, 5-HT promotes proliferation via 5-HTR1A, 5-HTR2B, and 5-HTR4, demonstrating functional consistency across receptor subtypes.^92^^,^^95^ In cholangiocarcinoma, activation of multiple receptors, including 5-HTR1A, 5-HTR2A, 5-HTR2B, 5-HTR4, and 5-HTR6 has been associated with enhanced tumor growth.^94^ This receptor redundancy and functional diversity suggest that single-receptor targeting may be insufficient to fully disrupt neurotransmitter-driven tumor progression. Instead, developing multi-receptor antagonists or combination strategies targeting multiple receptor subtypes may offer a more effective approach. Moreover, understanding the relative contribution of each receptor subtype to specific tumor-promoting processes (*e.g.*, proliferation, migration, immune evasion) will be critical for optimizing therapeutic selectivity and minimizing off-target effects.

More frequently, however, different receptors mediate distinct cellular functions. In hepatocellular carcinoma, for instance, GABAA signaling suppresses cell migration, while GABAB primarily inhibits proliferation.^14^^,^^87^ In breast cancer, activation of GABRA3 promotes migration and invasion, whereas GABAB can counteract norepinephrine-induced pro-migratory effects.^90^^,^^91^^,^^98^ Similarly, in breast cancer, 5-HTR2A activation enhances proliferation, whereas 5-HTR7 activation suppresses it.^93^^,^^99^ These observations suggest that tumors may selectively utilize specific neurotransmitter–receptor interactions depending on their progression stage or microenvironmental context, potentially through dynamic regulation of receptor expression. These functional divergences further underscore the need for receptor subtype-specific targeting in drug development. Broad inhibition of entire neurotransmitter pathways may lead to undesired effects by disrupting both pro- and anti-tumor signaling arms. Therefore, achieving high receptor selectivity will be critical for maximizing therapeutic efficacy while minimizing off-target effects.

Interestingly, the functional impact of a given neurotransmitter–receptor pair can differ across cancer types. For example, the GABA–GABAB axis regulates cell migration in prostate cancer, colon carcinoma, and breast cancer, but primarily controls proliferation in hepatocellular carcinoma.^87^, ^88^, ^89^, ^90^, ^91^ Likewise, 5-HT–5-HTR1A signaling suppresses proliferation in bladder cancer while promoting it in prostate cancer and cholangiocarcinoma.^92^^,^^94^^,^^96^ These observations underscore the context-dependent nature of neurotransmitter signaling in cancer, which may be shaped by differences in receptor abundance, downstream signaling networks, and tumor-specific cellular contexts. A deeper understanding of these variables could inform the development of more selective and effective neuromodulatory cancer therapies.

Notably, several tumor types appear to integrate signals from multiple neurotransmitter systems. In breast cancer, pathways involving GABA, norepinephrine, dopamine, 5-HT, and glutamate have all been implicated in tumor progression.^96^, ^97^, ^98^^,^^100^ Similar multimodal regulation has been reported in colon carcinoma (involving GABA, norepinephrine, 5-HT, and glutamate),^16^^,^^89^^,^^101^^,^^102^ as well as in pancreatic cancer, where GABA, norepinephrine, and glutamate signaling pathways converge.^103^, ^104^, ^105^ Whether these pathways are co-activated simultaneously or selectively engaged at different disease stages remains an open question. Understanding the regulatory mechanisms that determine receptor expression patterns and signaling specificity will be crucial for the development of effective neurotransmitter-targeted cancer therapies.

#### Neuropeptides

In addition to classical neurotransmitters, neuropeptides represent another crucial class of signaling molecules involved in nerve–tumor interactions. Neuropeptides are small, protein-like signaling molecules released by neurons that regulate a wide array of physiological functions. Unlike classical neurotransmitters, which typically mediate rapid, point-to-point synaptic transmission, neuropeptides act more slowly and diffusely.^106^^,^^107^ They often function as neuromodulators that shape long-term cellular responses and broader tissue-level signaling.^107^ Nonetheless, similar to neurotransmitters, neuropeptides engage in intercellular communication primarily through ligand–receptor interactions. A growing number of neuropeptides, including substance P (SP), neuropeptide Y (NPY), Bradykinin, calcitonin gene-related peptide (CGRP), vasoactive intestinal peptide (VIP), somatostatin, and gastrin-releasing peptide (GRP) (the mammalian homolog of amphibian bombesin), have been implicated in regulating tumor progression (Table 4).

**Table 4:** Functional roles of neuropeptide–receptor pathways in tumor progression.

Ligand	Receptor	Cancer type	Function	Reference
Substance P	TACR1	Breast cancer	Drives metastasis	109
	NK-1	Breast carcinoma	Induces migration	100
		Pancreatic cancer	Induces proliferation and invasion; promotes migration	108
		Lung cancer	Induces proliferation	305
Neuropeptide Y	Y1R	Breast cancer	Decreases cell proliferation	110
		Prostate cancer	Regulates proliferation	113
	Y2R	Breast cancer	Increases chemotaxis	111
		Cholangiocarcinoma	Antiproliferative effects	86
	Y5R	Breast cancer	Promotes proliferation and chemotaxis	111,112
Bradykinin	B2	Bladder cancer	Induces locomotory movement	117
CGRP	CLR	Oral squamous cell carcinoma	Promotes proliferation and migration	118
VIP	VIPR	Small-cell lung cancer	Stimulates proliferation	120
Somatostatin	SSTR5	Pancreatic endocrine tumor	Anti-proliferative action	115
	SSTR2	Pancreatic tumor	Inhibits proliferation	116
Bombesin	BBR	Small-cell lung cancer	Stimulates growth	120

Note: TACR1, tumoral tachykinin receptor 1; NK-1, neurokinin-1; CGRP, calcitonin gene-related peptide; CLR, CGRP receptor encoded by CALCRL; VIP, vasoactive intestinal peptide; VIPR, vasoactive intestinal peptide.

**Table 5:** Bidirectional crosstalk between nerves and TME components.

Nerve to TME				
Neural input	Target cell	Sub-type	Functional outcome	Reference
Perineural invasion	Immune cell	NK cell	Decreases infiltration	30
		Neutrophil	Increases infiltration	30
Serotonin	Immune cell	Macrophage	Modulation of polarization	134,135
		Dendritic cell	Immunomodulatory capacity	187
		T Cell	Regulates activation, proliferation, and function	141,143,144
		B Cell	Up-regulates the proliferation	306
		NK cell	Enhances the cytotoxicity and proliferation	177,178
	Stroma cell	CAF	CAF reprogramming	197
	Stroma cell	Endothelial cell	Vascular homeostasis	209,210
Norepinephrine	Immune cell	T Cells	Inhibits T cell proliferation	307
		B Cells	Enhances IgG production	181
		TAMs	Regulates TAM recruitment and polarization	133,137
		NK cells	Promotes expansion and effector function	176
		Dendritic cell	Regulates dendritic cell activation	137
		Myeloid-derived suppressor cell	Myeloid-derived suppressor cell recruitment	137
	Stroma cell	Endothelial cell	Vascular homeostasis	209
Epinephrine	Immune cell	Macrophages	Promotes M2 polarization	136
Acetylcholine	Immune cell	T Cells	Impairs CD4^+^ T cell differentiation	156
		B Cells	Promotes maturation	182
Substance P	Immune cell	T Cells	Modulates adhesion and proliferation	158,159
Neuropeptide Y	Immune cell	T Cells	Induces T cell adhesion	159
Somatostatin	Immune cell	T Cells	Induces T cell adhesion	159
CGRP	Immune cell	T Cells	Induces T cell adhesion; decreases γδ T cell numbers	159,184
		Neutrophils	Inhibits recruitment	184,185
Dopamine	Immune cell	T Cell	Induces T cell adhesion	159
Endorphin	Immune cell	Mononuclear cell	Stimulates chemotaxis	308
Enkephalin	Immune cell	Mononuclear cell	Stimulates chemotaxis	308
Stress	Immune cell	NK cell	Enhances cytotoxicity	175
NGF	Immune cell	Mast cell	Increases the size and the number	309
		T Cell	Promotes proliferation	167
		B Cell	Promotes proliferation and differentiation	167,168
		Polymorphonuclear leukocyte	Promotes recruitment	310,311
		Monocyte	Induces differentiation	312
*TME to nerve*				

Source cell	Sub-type		Functional outcome	Reference

Immune cell	T Cell		Promotes NGF synthesis and release	145
	Macrophage		Induces NGF production via IL-1β signaling	313
	TAM		Promotes perineural invasion	138, 139, 140
	EMΦ		Secretes high levels of GDNF, promotes perineural invasion	124
	T Cell		Releases acetylcholine	314
	B Cell		Releases acetylcholine	183,314
Stroma cell	Cancer-associated fibroblast		Enhances neural remodeling through SLIT2 signaling	200
	Cancer-associated fibroblast		Promotes perineural invasion	133,190,196
	Cancer-associated fibroblast		Contributes to Schwann-cell-induced axonal growth	201

Note: TME, tumor microenvironment; EMΦ, a subpopulation of microglia/macrophage; NK cell, natural killer cell; TAM, tumor-associated macrophage; NGF, nerve growth factor; GDNF, glial cell line-derived neurotrophic factor; CGRP, calcitonin gene-related peptide.

Among them, SP, an 11-amino acid neuropeptide belonging to the tachykinin family, exerts its biological effects primarily via the neurokinin-1 receptor (NK1R).^108^ SP has been reported to promote tumor cell migration in breast carcinoma and pancreatic cancer, and to enhance tumor cell proliferation in pancreatic and lung cancers, highlighting its role in both local invasion and growth.^100^^,^^108^ Notably, a recent study demonstrated that SP can also promote breast cancer metastasis through direct binding to tumoral tachykinin receptors (TACR1), further supporting its pro-metastatic function within the TME.^109^

NPY, a 36-amino acid peptide abundantly expressed in both the central and peripheral nervous systems, binds to multiple G protein-coupled receptors, including Y1, Y2, Y4, and Y5 receptors. In breast cancer, NPY displays receptor-dependent functional diversity: binding to Y1R suppresses estrogen-induced cell proliferation, whereas binding to Y5R enhances proliferation.^110^, ^111^, ^112^ Both Y2R and Y5R have been associated with increased cancer cell migration, suggesting a complex regulatory role in tumor behavior.^111^^,^^112^ The functional impact of NPY signaling is further modulated by tumor type and intracellular context. In prostate cancer, the effect of NPY–Y1R interaction on proliferation is dependent on the temporal dynamics of MAPK activation.^113^ In cholangiocarcinoma, NPY binding to Y2R has been shown to suppress tumor cell proliferation and invasion by inhibiting intracellular d-myo-inositol 1,4,5-trisphosphate and PKCα signaling pathways.^86^

Beyond SP and NPY, several additional neuropeptides have been implicated in modulating tumor progression through diverse receptor-mediated mechanisms. These molecules, while studied to varying extents, collectively highlight the breadth of neuropeptide-driven influence on cancer biology. Somatostatin is another well-characterized neuropeptide, existing in two bioactive forms of 14 and 28 amino acids.^114^ It acts through five somatostatin receptor subtypes (SSTR1–SSTR5), each with distinct expression patterns and functions. In pancreatic endocrine tumors, somatostatin inhibits cell proliferation primarily via SSTR5,^115^ whereas in pancreatic ductal adenocarcinoma, its antiproliferative effect is mainly mediated through SSTR2,^116^ underscoring the receptor-context specificity of its action. Bradykinin, a nonapeptide generated from kininogen precursors via kallikrein cleavage, also plays a role in tumor-related processes. In bladder cancer, bradykinin has been shown to promote cell motility through activation of the B2 receptor,^117^ suggesting a role in facilitating local invasion. CGRP is a 37-amino acid peptide derived from alternative splicing of the calcitonin gene. It binds to the CGRP receptor complex and has been implicated in promoting both tumor growth and angiogenesis in Lewis lung carcinoma,^118^ reflecting its contribution to the vascular remodeling commonly seen in aggressive tumors. Vasoactive intestinal peptide (VIP), composed of 28 amino acids, signals through VPAC1 and VPAC2 receptors.^119^ Upon receptor activation, VIP has been demonstrated to stimulate proliferation in neuroendocrine tumors, particularly small-cell lung carcinoma,^120^ supporting its role in the growth of tumors with neuroendocrine features. Similarly, GRP, the mammalian homolog of amphibian bombesin and a member of the bombesin-like peptide family, acts through bombesin receptors (BBRs) to promote the proliferation of neuroendocrine tumors, including small-cell lung cancer.^120^ Its functional similarity to VIP in promoting neuroendocrine tumor growth further highlights the relevance of bombesin-like peptides in cancer biology.

Together, these findings illustrate that neuropeptides exert multifaceted and tumor-specific effects through distinct receptor-mediated pathways. Their pleiotropic roles in proliferation, migration, invasion, and angiogenesis make them compelling targets for further investigation in the context of nerve–tumor crosstalk.

#### Organelle transfer

Recent studies show that neurons transfer mitochondria to neighboring cancer cells via contact-dependent structures consistent with tunneling nanotubes.^121^ This transfer enhances tumor bioenergetics, metabolic plasticity, and survival, especially during metastasis, and denervation or interruption of neural input diminishes these advantages in coculture and *in vivo* models.^121^ These findings elevate direct nerve–tumor contact to a core crosstalk axis alongside neurotransmitters.

### Cancer-driven neural modulation

While much attention has been given to how neural inputs regulate tumor progression through neurotransmitters, neuropeptides, and structural innervation, emerging evidence highlights that cancer cells, in turn, actively remodel and influence the nervous system. This bidirectional crosstalk enables tumors to co-opt neural elements to support their own growth and survival. In this section, we explore how tumors affect nerves.

A primary mechanism by which cancer cells modulate nerves is through the secretion of neurotrophic factors, such as NGF, BDNF, and glial cell line-derived neurotrophic factor (GDNF), which stimulate axonal sprouting and enhance neural infiltration.^122^, ^123^, ^124^ As mentioned above, this tumor-induced remodeling has been widely observed in cancers like pancreatic and prostate cancer, where increased nerve density correlates with aggressiveness and poor prognosis.^28^^,^^39^ Beyond supporting tumor progression, newly formed innervation contributes to PNI and neural damage, often exacerbating cancer-associated pain.

Beyond structural remodeling, tumor-derived factors can induce functional and phenotypic changes in neurons. Increasing evidence shows that tumors modulate neuronal gene expression, leading to neurotransmitter switching, altered ion channel activity, and reorganization of autonomic innervation.^125^^,^^126^ A well-documented example is the autonomic shift observed in prostate cancer: sympathetic nerves are enriched in early tumorigenesis and promote initial tumor growth, while parasympathetic nerves become predominant in later stages and facilitate invasion and metastasis.^125^ This dynamic shift in the sympathetic–parasympathetic balance represents a form of tumor-induced neuronal reprogramming.^125^

Moreover, recent studies have revealed that certain tumors can reprogram sensory neurons into pro-tumorigenic states. For example, in head and neck squamous cell carcinoma, loss of p53 in tumor cells has been shown to induce transcriptional reprogramming in neighboring sensory neurons, resulting in a phenotype that actively supports tumor progression.^126^ These reprogrammed neurons may exhibit altered excitability, enhanced secretion of neuroactive factors, and modulation of the local immune microenvironment, suggesting that neurons, far from being passive components, can be co-opted into active contributors to the tumor niche.^126^

Together, cancer-driven neural modulation represents a critical and evolving aspect of tumor biology. By secreting neurotrophic factors, altering neuronal gene expression, and reprogramming neural subtypes, tumors not only recruit structural innervation but also reshape neural function to favor tumor growth, invasion, and immune evasion. These findings redefine nerves as active participants in the TME rather than passive conduits.

## Neural regulation of tumor immune cells

Beyond directly shaping tumor cell behavior, neural signaling exerts profound effects on the TME (Table 5). A growing body of evidence indicates that nerves and their associated neurotransmitters and neuropeptides modulate the activity and function of various tumor-infiltrating immune cells, including tumor-associated macrophages (TAMs), T cells, neutrophils, natural killer (NK) cells, and B cells, thereby contributing to immune evasion and tumor progression.^29^^,^^32^^,^^42^^,^^69^^,^^78^^,^^127^, ^128^, ^129^, ^130^, ^131^ This neuro-immune crosstalk represents a critical, yet underappreciated, dimension of tumor–host interactions, with emerging implications for immunotherapy resistance and therapeutic targeting. In the following sections, we detail how specific neural pathways shape the function of individual immune cell subsets within the TME.

### TAMs

TAMs constitute a major component of the TME and play critical roles in tumor initiation, progression, and the establishment of an immunosuppressive TME.^132^ The interaction between the nervous system and TAMs represents a pivotal regulatory axis in the modulation of tumor immunity. Through the release of neurotransmitters, neurotrophic factors, and other bioactive signals, nerves influence the recruitment, polarization, and function of TAMs, thereby contributing to the establishment and maintenance of an immunosuppressive TME.^133^, ^134^, ^135^, ^136^, ^137^, ^138^ In breast cancer, epinephrine promotes the polarization of macrophages toward the M2 phenotype by engaging β-adrenergic receptor 2 (ADRβ2) on TAMs, facilitating tumor progression.^136^ Although β-adrenergic signaling may not significantly enhance primary tumor growth, it markedly promotes macrophage recruitment and M2-like differentiation, ultimately driving metastatic dissemination.^133^

Similarly, 5-HT interacts with its receptor 5-HT7 to suppress the release of pro-inflammatory cytokines from macrophages.^134^ During the monocyte-to-macrophage differentiation process, blocking 5-HT2B and 5-HT7 receptors simultaneously promotes macrophage polarization toward the M2 phenotype.^134^ Mechanistically, 5-HT binding to 5-HT2B activates the aryl hydrocarbon receptor (AhR) signaling cascade, up-regulating downstream anti-inflammatory genes and reinforcing an immunosuppressive macrophage phenotype.^135^ In addition to individual neurotransmitter-mediated effects, broader neural circuits also shape macrophage polarization. For example, the sympathetic nervous system exerts systemic immunomodulatory effects through the release of catecholamines, which promote the polarization of TAMs toward an M2-like phenotype.^137^ Such findings underscore that both localized neurotransmitter signaling (*e.g.*, via epinephrine and serotonin) and systemic neural input act in concert to orchestrate TAMs’ function and reinforce tumor immune evasion.

Reciprocally, TAMs can also exert significant effects on nerve structures within the TME, particularly in the context of PNI (Table 5). In pancreatic ductal adenocarcinoma, high TAM infiltration correlates with PNI positivity, implicating TAMs in the facilitation of nerve invasion.^138^^,^^139^ One mechanism involves the activation of endoneurial macrophages by pancreatic ductal adenocarcinoma cells, which results in elevated secretion of GDNF.^124^ GDNF, in turn, promotes PNI by activating the RET/ERK signaling pathway in tumor cells.^124^ Another reported pathway involves matrix metalloproteinase-1 (MMP1), which activates AKT signaling in dorsal root ganglia, leading to the expression of SP via protease-activated receptor-1 (PAR1).^140^ SP subsequently enhances the migratory and invasive capacity of NK1R-expressing pancreatic ductal adenocarcinoma cells, further driving PNI.^140^

This bidirectional communication between nerves and TAMs underscores their cooperative roles in shaping the TME. Nerve-derived signals modulate TAMs’ recruitment and functional polarization, establishing a pro-tumorigenic niche. In return, TAMs actively participate in neural remodeling and invasion, facilitating tumor progression through neuro-immune signaling networks. Elucidating these complex interactions presents promising opportunities for the development of novel therapeutic interventions targeting the nerve–TAMs axis in cancer.

### T cells

In parallel with TAMs, T cells engage in dynamic crosstalk with nerves within the TME, which plays a pivotal role in orchestrating tumor progression, immune modulation, and therapy resistance.^141^^,^^142^ Neural signaling regulates T cell activation, differentiation, and effector function through a range of neurotransmitters and neuropeptides, thereby contributing to immune suppression or stimulation depending on context.^141^^,^^143^^,^^144^ This communication is increasingly recognized as bidirectional, as T cells themselves can also influence neural remodeling and functional plasticity within the TME.^145^, ^146^, ^147^ Such neuro-immune interactions collectively shape the balance between immune surveillance and immune evasion in cancer.

Neurotransmitter signaling plays a pivotal role in the interaction between nerves and T cells. For instance, 5-HT is considered a cofactor in T cell activation.^144^ Naive T cells predominantly express the 5-HT7 receptor, and signaling through this receptor contributes to the early stages of T cell activation.^143^^,^^144^ As activation progresses, T cells begin to express additional 5-HT receptors, including 5-HT1B and 5-HT2A, expanding their responsiveness to serotonergic signaling.^143^^,^^144^ Moreover, stimulation of the 5-HT3 receptor with 2-methyl-5-HT accelerates T cell proliferation by facilitating cell cycle progression from the S to G2/M phase.^143^ At later stages of activation, effector T cells up-regulate 5-HT2 receptors, and 5-HT engagement of these receptors enhances inflammatory cytokine secretion, thereby amplifying pro-inflammatory responses.^141^ However, 5-HT’s effects on T cells are not uniformly pro-inflammatory. While it generally promotes T cell differentiation and cytotoxic cytokine production, studies in murine models of pancreatic and colorectal cancer have paradoxically demonstrated that reduction of peripheral 5-HT levels enhances T cell infiltration and differentiation within tumors.^142^ Mechanistically, this effect may be attributed to the serotonin transporter (SERT), which acts as a negative regulator of CD8^+^ T cell-mediated immunity by depleting autocrine serotonin in the TME.^148^ Notably, combining peripheral 5-HT inhibition with immune checkpoint blockade therapy increases the intra-tumoral abundance of cytotoxic CD8^+^ T cells and reduces immunosuppressive myeloid-derived suppressor cells, leading to significantly improved therapeutic outcomes.^142^ These context-dependent effects of 5-HT highlight the complexity of neurotransmitter–immune interactions. Elucidating the molecular mechanisms underlying this variability will be essential for harnessing the 5-HT pathway as a therapeutic target in cancer immunotherapy.^149^

The role of epinephrine and norepinephrine in modulating T cell function and cancer progression has garnered increasing research attention. Emerging evidence indicates that elevated epinephrine levels, such as those induced by physical exercise, can promote T cell infiltration into tumors, thereby augmenting the efficacy of cancer immunotherapies.^150^ Conversely, norepinephrine exerts immunosuppressive effects by activating beta2-adrenergic receptors (β2-ARs) on T cells, which in turn suppress the production of pro-inflammatory cytokines like IFN-γ and facilitate the differentiation of regulatory T cells (Tregs), contributing to the development of an immunosuppressive TME.^151^^,^^152^ Notably, pharmacologic inhibition of β2-ARs has been shown to enhance T cell activation, proliferation, and cytokine production, suggesting its potential as an immunomodulatory strategy.^153^ The combination of β2-AR blockade with chimeric antigen receptor T cell therapy has yielded promising results in preclinical models, offering a compelling rationale for integrated therapeutic approaches.^153^ Together, these findings underscore the dual and context-dependent roles of epinephrine and norepinephrine in shaping T cell immunity through β-adrenergic signaling, and highlight the importance of finely tuned adrenergic modulation to optimize immunotherapeutic efficacy.

Another key neurotransmitter system shaping T cell responses is cholinergic signaling. In pancreatic ductal adenocarcinoma, PNI leads to elevated levels of acetylcholine (ACh) within the TME, which in turn suppresses C–C motif chemokine ligand 5 (CCL5) expression in tumor cells and impairs CD8^+^ T cell recruitment.^154^ In addition to these indirect effects, ACh directly inhibits IFN-γ production by CD8^+^ T cells and enhances the differentiation of CD4^+^ T cells into the Th1 subset.^154^ Mechanistically, blocking the M1 muscarinic ACh receptor on CD8^+^ T cells, via either genetic deletion or pharmacological inhibition, significantly impairs their cytotoxic differentiation.^155^ Moreover, acetylcholine activation of α7 nicotinic acetylcholine receptors (α7 nAChRs) on CD4^+^ T cells has been shown to promote the differentiation of naïve CD4^+^ T cells into both immunosuppressive Tregs and cytotoxic effector T cells.^156^ This dual regulatory capacity likely contributes to the limited therapeutic efficacy of α7 nAChR agonists observed in both preclinical and clinical settings. Together, these findings underscore the complexity and context dependence of cholinergic signaling in modulating T cell responses within the TME.

Beyond classical neurotransmitters, neuropeptides, including SP, CGRP, and NGF, also exert important regulatory effects on T cells within the TME. SP has been shown to promote T cell proliferation and enhance interleukin-2 (IL-2) expression in activated T cells.^157^^,^^158^ Additionally, SP also participates in the regulation of integrin-mediated T-cell adhesion, where blocking SP enhances T-cell adhesion.^159^ The role of SP in regulating Tregs appears to be context-dependent. In dry eye disease, elevated SP levels reduce Treg frequencies and impair their suppressive function; conversely, antagonizing its receptor, NK-1R, restores Treg function and mitigates the pathogenic Th17 response.^160^ However, in the setting of cardiac ischemia–reperfusion injury, SP exhibits the opposite effect, increasing both IL-10 levels and circulating Tregs.^161^ In the context of cancer, SP has been implicated in promoting the progression of breast carcinoma and pancreatic cancer. However, its direct effects on intra-tumoral T cells remain poorly understood and warrant further investigation.^100^^,^^108^

In addition to SP, CGRP has likewise emerged as a key modulator of T cell function within the immunosuppressive TME. In medullary thyroid cancer, elevated CGRP levels are associated with reduced functional activity of tumor-infiltrating T cells.^162^ In head and neck squamous cell carcinoma, CGRP has been shown to directly suppress CD8^+^ T cell activation, thereby impairing anti-tumor immunity.^163^ In melanoma, CGRP exacerbates CD8^+^ T cell exhaustion, facilitating immune evasion by the tumor.^85^ Conversely, loss of CGRP function appears to restore antitumor immunity. Knockout of CGRP in oral cancer models leads to increased infiltration of both CD4^+^ and CD8^+^ T cells, enhancing intra-tumoral immune responses.^164^ Additionally, activation of transient receptor potential cation channel subfamily V member 1-positive (TRPV1^+^) neurons in the dorsal root ganglia promotes CGRP release, which in turn reduces Treg numbers in the colon and cecum, suggesting a complex role of CGRP in shaping immune balance across tissues.^165^ Clinically, several CGRP-targeting agents, including eptinezumab, galcanezumab, erenumab, and ubrogepant, are currently approved for migraine management.^166^ However, whether these agents can be repurposed as immunomodulatory therapies in oncology, either alone or in combination with immune checkpoint blockade (*e.g.*, anti-PD-1/PD-L1), remains an important avenue for future investigation.

NGF represents another neuropeptide that plays a crucial role in T cell biology by influencing their proliferation, differentiation, and function within the TME.^167^^,^^168^ Notably, a subset of CD4^+^ T cells in both humans and mice expresses NGF along with its cognate receptors, TrkA and p75NTR.^145^, ^146^, ^147^ This expression enables these cells to regulate their own differentiation into specific subsets via autocrine NGF–TrkA/p75NTR signaling.^145^, ^146^, ^147^ In addition to autocrine regulation, NGF-producing CD4^+^ T cells can also promote axonogenesis in the surrounding tissue through paracrine mechanisms, further highlighting their role in neuroimmune crosstalk.^122^^,^^169^^,^^170^ This dual capacity of NGF to influence both immune and neural components suggests its potential involvement in tumor progression and immune evasion. In hepatocellular carcinoma, the NGF–NGFR pathway contributes to resistance to anti-PD-1 immunotherapy.^171^ Similarly, in melanoma, activation of the NGF–TrkA axis facilitates immune evasion and suppresses effective anti-tumor immunity.^172^ Conversely, inhibition of this pathway not only sensitizes tumors to immune checkpoint blockade but also promotes the activation of long-lived, low-affinity memory T cells, thereby sustaining durable anti-tumor responses.^172^ Collectively, these findings position NGF as a key regulator of T cell function and neuroimmune dynamics, with significant implications for cancer immunotherapy.

### NK cells

NK cells are pivotal components of the innate immune system, known for their ability to recognize and eliminate tumor cells without prior sensitization.^173^ The crosstalk between the nervous system and NK cells plays an important role in shaping anti-tumor immunity.^30^^,^^174^^,^^175^ In murine models of pancreatic and lung cancer, activation of β-adrenergic signaling has been shown to enhance NK cell infiltration and cytotoxicity within the TME, thereby suppressing tumor growth.^175^

Similarly, during viral infection, NK cells up-regulate Adrb2, the gene encoding the β2-adrenergic receptor, and conditional deletion of this receptor in NK cells impairs their proliferation and effector function, underscoring the importance of adrenergic signaling in NK cell biology.^176^ However, whether the TME modulates Adrb2 expression in NK cells remains largely unexplored. Serotonin also modulates NK cell activity. It enhances NK cell cytotoxicity via serotonin receptor activation, particularly in the presence of monocytes.^177^ Furthermore, long-term treatment with selective serotonin reuptake inhibitors in patients with major depressive disorder has been linked to increased NK cell populations in peripheral blood,^178^ suggesting a potential role for serotonergic signaling in enhancing NK cell-mediated immunity.

However, in tumors exhibiting PNI, such as intrahepatic cholangiocarcinoma, NK cell infiltration is notably reduced, suggesting that nerve involvement may hinder NK cell–mediated immune responses.^30^ Interestingly, in the context of peripheral nerve injury, NK cells are capable of infiltrating damaged nerves within days, where they contribute to axonal degeneration and nerve remodeling.^174^ These observations imply that nerve-derived signals may exert context-dependent effects on NK cell function. Targeting these pathways to restore NK cell activity in nerve-rich tumor niches, particularly in PNI-positive cancers, may represent a novel therapeutic avenue.

### B cells

Advances in single-cell sequencing and spatial transcriptomics have brought renewed attention to the role of B cells in the TME.^179^^,^^180^ Emerging evidence suggests that the nervous system can regulate various aspects of B cell biology, including their proliferation, differentiation, and immunoglobulin class switching.^109^^,^^110^^,^^181^^,^^182^ For instance, splenic B cells express receptors for NGF, allowing NGF to directly modulate their proliferation and differentiation.^167^^,^^168^ In addition, B cells express muscarinic acetylcholine receptors (mAChRs), which are involved in cytokine production and facilitate the differentiation of B cells into plasma cells, supporting the isotype switching from IgM to IgG.^181^^,^^182^ Interestingly, B cells are not only targets but also an important source of the neurotransmitter ACh.^183^ ACh-producing B cells can suppress peritoneal neutrophil recruitment in an autonomously regulated manner, independent of vagal nerve input.^183^ These findings reveal a previously underappreciated role of B cells as active participants in neuroimmune communication. Despite these insights, the direct interplay between nerves and B cells within the TME remains poorly characterized. Given the increasing recognition of B cells as both immune effectors and organizers of tertiary lymphoid structures in cancer, a deeper understanding of their neuroregulation may open new avenues for therapeutic intervention in solid tumors.

### Neutrophils

In intrahepatic cholangiocarcinoma, patients exhibiting PNI often show elevated neutrophil infiltration, which correlates with poorer overall survival.^30^ However, the mechanisms underlying this association remain largely undefined. Interestingly, the neurotransmitter ACh has been shown to suppress local neutrophil recruitment under certain conditions.^183^ Likewise, during bacterial infections, TRPV1^+^ nociceptor neurons limit neutrophil recruitment and immune surveillance by releasing the neuropeptide calcitonin gene-related peptide (CGRP).^184^^,^^185^ In cancer, the paradoxical coexistence of PNI and increased neutrophil infiltration raises important questions about the underlying mechanisms. These findings suggest that neural signaling can exert either pro- or anti-inflammatory effects on neutrophil behavior, depending on the context. Deciphering how PNI enhances neutrophil recruitment could provide key insights into neuroimmune crosstalk in the TME and its contribution to disease progression.

### Dendritic cells (DCs)

Nerve–tumor interactions play an important role in modulating DCs’ behavior, influencing their development, activation, and capacity to elicit anti-tumor immune responses.^137^^,^^186^

In medullary thyroid carcinoma, aberrant expression of CGRP has been associated with impaired DC development, characterized by enhanced cyclic AMP (cAMP) signaling and up-regulation of the transcription factor Kruppel-like factor 2 (KLF2).^162^ Notably, pharmacological blockade of the CGRP receptor can reverse these defects *in vitro*, suggesting a therapeutic opportunity to restore DCs’ function.^162^ Beyond tumor-derived neuropeptides, the autonomic nervous system also contributes to DCs’ modulation. Vagus nerve stimulation has been shown to inhibit the expansion of myeloid-derived suppressor cells and enhance DCs’ function via ACh signaling through the α7 nicotinic acetylcholine receptor (α7nAChR), thereby promoting anti-tumor immunity in animal models.^186^ Similarly, adrenergic signaling influences DCs’ activity: modulation of catecholamine levels reduces myeloid-derived suppressor cell accumulation while facilitating DCs activation, further supporting effective anti-tumor responses.^137^ In addition, 5-HT has been implicated in the regulation of DCs’ function under inflammatory conditions. Human monocyte-derived CD1a^+^ DCs express the 5-HT2B receptor, and activation of this receptor suppresses TLR2-, TLR3-, and TLR7/8-induced production of pro-inflammatory cytokines and chemokines, including TNF-α, IL-6, IL-8, IP-10, and IL-12.^187^ Interestingly, this suppressive effect does not extend to type I interferon-β responses, indicating that serotonergic signaling selectively modulates DC activation depending on the immune context.^187^ Collectively, these findings highlight the multifaceted role of neural signaling in regulating DCs’ biology, with implications for immune activation, tumor immune evasion, and therapeutic intervention within the TME.

In summary, neural regulation of immune cells within the TME constitutes an emerging frontier in cancer research with considerable translational potential. Advancing this field will require a deeper understanding of the molecular and cellular mechanisms through which neural signals, such as neurotransmitters, neuropeptides, and neurotrophic factors, modulate immune cell behavior within tumors. Elucidating these pathways is essential for identifying actionable targets and developing neuromodulatory strategies capable of selectively disrupting pro-tumorigenic nerve–immune interactions. Such interventions, whether pharmacologic, genetic, or device-based, hold promise for enhancing anti-tumor immunity. As our knowledge of neuroimmune crosstalk deepens, these insights may enable the design of innovative therapeutic approaches that synergize with existing immunotherapies and ultimately improve clinical outcomes.

## Neural remodeling of tumor stroma

The interplay between the nervous system and tumor stromal cells represents a critical and evolving dimension of tumor biology. Key stromal components, including cancer-associated fibroblasts (CAFs) and endothelial cells, help shape the TME and are essential for supporting tumor growth, angiogenesis, immune evasion, and metastatic spread.^188^^,^^189^ Increasing evidence indicates that infiltrating nerve fibers communicate with these stromal cells through neurotransmitters, neuropeptides, and growth factors, thereby influencing their function and reinforcing a tumor-promoting niche.^123^^,^^190^^,^^191^ Understanding the mechanisms underlying nerve–stroma crosstalk may reveal novel therapeutic vulnerabilities and inform the development of next-generation cancer treatments that target the neuro–stromal axis.

### Cancer-associated fibroblasts

Cancer-associated fibroblasts (CAFs) are a major component of the TME and play a critical role in tumor progression, immune modulation, and therapy resistance.^192^^,^^193^ Their functional significance stems from their ability to interact dynamically with cancer cells, immune populations, the extracellular matrix (ECM), and infiltrating nerves.^194^^,^^195^ The bidirectional communication between CAFs and nerves is increasingly recognized as a critical driver of PNI and axonogenesis in diverse malignancies.^190^^,^^191^^,^^196^^,^^197^

In several cancers, including head and neck squamous cell carcinoma and prostate cancer, elevated CAF density has been associated with a higher incidence of PNI.^190^^,^^196^^,^^198^^,^^199^ Building on these observations, growing evidence indicates that CAFs actively contribute to both PNI and axonogenesis through multiple mechanisms. For example, in prostate cancer, CAFs promote PNI by up-regulating YAP1 signaling in cancer cells,^191^ while in oral cavity squamous cell carcinoma, they promote nerve invasion through matrix metalloproteinase-2 (MMP-2).^196^ In pancreatic cancer, CAFs facilitate PNI through extracellular vesicle-mediated delivery of a PNI-associated transcript (PIAT) and promote axonogenesis by stimulating sympathetic nerve outgrowth.^198^^,^^199^ CAF-derived SLIT2, a neuronal guidance cue, has been shown to induce neurite outgrowth from dorsal root ganglia neurons and to promote Schwann cell migration and proliferation through N-cadherin/β-catenin signaling; notably, inhibition of the SLIT2/ROBO axis can disrupt these stromal–neural interactions.^200^ Furthermore, ephrin-B/EphB2 signaling from fibroblasts plays a complementary role by guiding Schwann cell migration from nerve stumps, thereby facilitating axonal regeneration.^201^

In addition to being influenced by CAF-derived signals, nerves can also modulate the phenotype of CAFs, reprogramming them into tumor-promoting subtypes. In pancreatic cancer, Schwann cell-derived interleukin-1α drives the transformation of CAFs into inflammatory CAFs (iCAFs), which in turn amplify tumor-supportive inflammation.^202^ Neurotransmitters also modulate CAF plasticity; in colorectal cancer, 5-HT induces serotonylation of histone H3 at glutamine 5 (H3Q5ser) in CAFs, driving their transition into an iCAF-like state that promotes tumor proliferation, invasion, and macrophage polarization.^197^ Targeting this epigenetic modification by silencing the serotonin transporter SLC22A3 or inhibiting transglutaminase 2 (TGM2) mitigates these tumor-promoting effects.^197^ Collectively, these findings underscore the reciprocal interactions between CAFs and nerves in shaping a pro-tumorigenic microenvironment. Targeting the CAF–nerve axis offers a promising strategy for disrupting PNI, axonogenesis, and stromal reprogramming in cancer.

### Endothelial cells

Endothelial cells (ECs) are a critical component of the TME, contributing to tumor progression through their roles in angiogenesis, immune evasion, metastasis, and therapy resistance.^203^^,^^204^ In addition to supplying oxygen and nutrients, angiogenesis facilitates tumor–nerve interactions by providing a scaffold for Schwann cell bundles and axonal growth, thereby promoting PNI and axonogenesis.^22^^,^^205^

ECs contribute to tumor-associated neural remodeling through multiple mechanisms, including the release of neurotrophic factors and extracellular vesicles. For example, BDNF released by ECs significantly enhances axonal outgrowth in peripheral nerves.^123^ Additionally, ECs support the proliferation and migration of Schwann cells after injury,^206^ laying the groundwork for axonal extension and facilitating tumor-associated nerve remodeling. EC-derived exosomes also play an important role in this process: they deliver miR-199a-5p to activate PI3K/AKT/PTEN signaling pathways in Schwann cells, promoting axonal regeneration, myelination, and functional recovery.^207^ Similarly, exosomes from NTN1^+^ ECs deliver let-7a-5p to create a pro-regenerative niche conducive to axon repair.^208^ Conversely, under hypoxic conditions, ECs undergo reciprocal modulation by neural signals; they increase glycolytic activity in response to SC-derived exosomes carrying miR-21-5p, thereby enhancing intraneural revascularization and further facilitating neural remodeling.^205^ This reciprocal interaction may constitute a positive feedback loop in tumors, exacerbating PNI and axonogenesis, particularly under hypoxic or injury-associated conditions. Disrupting this loop could represent a promising therapeutic strategy to mitigate tumor-associated neural remodeling.

This bidirectional crosstalk extends beyond structural remodeling: just as ECs modulate neural growth, neural-derived signals in turn profoundly influence endothelial cell function within the TME. Neurotransmitters and neuropeptides exert multifaceted regulatory effects on ECs’ function. Among neurotransmitters, ACh reduces the expression of endothelial adhesion molecules via muscarinic receptors, thereby modulating immune cell trafficking.^183^ Norepinephrine (NE) helps maintain endothelial barrier integrity,^209^ whereas 5-HT enhances EC proliferation through activation of 5-HT2 receptors.^209^^,^^210^ Neuropeptides also promote EC activation. SP stimulates ECs’ proliferation and angiogenesis, facilitating tumor metastasis in thyroid cancer.^211^ NGF promotes EC migration via TrkA signaling and, under inflammatory conditions, further enhances EC proliferation and adhesion molecule expression, thus promoting immune cell recruitment to the endothelium.^212^^,^^213^ These findings underscore the diverse and complex interactions between nerves and endothelial cells, suggesting that targeting and disrupting these interactions could provide a novel approach for treating tumors with neural involvement.

## Targeting nerve–tumor interactions

Given the critical roles of nerve-driven processes such as PNI, axonogenesis, and neurogenesis in tumor progression and metastasis, targeting these interactions offers substantial potential to improve cancer treatment outcomes. Extensive preclinical research on nerve–tumor crosstalk has laid the foundation for therapeutic approaches that not only target cancer cells directly but also disrupt the neural circuits that support tumor progression.^85^, ^86^, ^87^^,^^214^ Therapeutic strategies have emerged to modulate nerve–tumor signaling, ranging from inhibition of neurotransmitters to repurposing neuroactive drugs and ablating nerve structures.^125^^,^^214^^,^^215^ This section outlines current strategies, ongoing challenges, and future directions for targeting nerve–tumor interactions in cancer therapy.

Among available approaches, the most direct way to disrupt nerve–tumor connections is direct ablation of peripheral nerves. In preclinical settings, surgical or chemical denervation, such as sympathectomy, vagotomy, and selective sensory fiber ablation, as well as genetic or chemogenetic silencing, consistently suppresses tumor initiation, growth, invasion, and perineural spread.^126^^,^^165^^,^^216^^,^^217^ These findings establish a causal contribution of innervation. In clinical practice, nerve-directed procedures are used mainly for palliation. Endoscopic-ultrasound-guided celiac plexus neurolysis or splanchnicectomy in pancreatic cancer reproducibly reduces pain and opioid use but has not shown a survival benefit and carries procedure-specific risks, including orthostatic hypotension, diarrhea, and neuropathic pain, as well as variable durability due to re-innervation.^218^, ^219^, ^220^ Taken together, direct ablation underscores the therapeutic relevance of neural circuits and is presently best positioned as a supportive adjunct within multimodal care. Future studies should emphasize more selective, anatomy-guided targeting and non-destructive neuromodulation, coupled with mechanistic endpoints, including changes in innervation density and in immune and stromal states, to balance efficacy and safety.

Another promising area involves targeting neurotransmitter pathways. A number of neurotransmitter-modulating drugs, some of which are already clinically approved for the treatment of psychiatric disorders and mood disturbances, have entered cancer-related clinical trials (Table 6). For example, antipsychotic agents such as thioridazine, chlorpromazine, trifluoperazine, fluspirilene, pimozide, and penfluridol exert their effects primarily through antagonism of dopamine receptors.

**Table 6:** Neuroactive drugs with anti-tumor potential in clinical trials.

Drug	Type	Mechanism of action	Clinical trial No.	Phase	Reference
Thioridazine	Dopamine receptor D2 antagonist	Induces apoptosis	NCT02307396	Phase Ⅳ	215
Valproic acid	Anti-epileptic	Induces apoptosis; decreases invasion and migration	NCT00186186	Phase Ⅳ	315
Fluoxetine	Selective serotonin reuptake inhibitor	Reduces proliferation and induces apoptosis; enhances paclitaxel efficacy	NCT05458479	Phase Ⅳ	315
Propranolol	β-blocker	Reduces angiogenesis; enhances paclitaxel and 5-fluorouracil efficacy	NCT01908972	Phase Ⅳ	316
Mirtazapine	NaSSA	Enhances chemotherapy efficacy by modulating the tumor microenvironment	NCT04155008	Phase Ⅳ	315
Penfluridol	Anti-psychotic	Inhibits tumor growth and metastasis in TNBC; suppresses migration and invasion	NCT01655680	Phase Ⅱ	227
Pimozide	Anti-psychotic	Induces apoptosis; disrupts angiogenesis; reduces migration and invasion; suppresses fibroblast-to-myofibroblast differentiation	NCT00158223	Phase Ⅳ	226
Fluspirilene	CDK2 inhibitor	Enhances therapeutic efficacy combined with 5-fluorouracil	NCT00119509	Phase Ⅳ	225
Promethazine	Anti-histamine	Inhibits tumor growth and induces apoptosis	NCT02648490	Phase Ⅰ	317
Trifluoperazine	Anti-psychotic	Induces apoptosis; enhances the effects of 5-fluorouracil and oxaliplatin	NCT02704962	Phase Ⅳ	223,224
Chlorpromazine	Anti-psychotic	Inhibits proliferation and induces apoptosis	NCT00169039	Phase Ⅳ	221,222
Desipramine	TCA	Enhances the efficacy of platinum-based therapies	NCT00166114	Phase Ⅳ	230
Imipramine	TCA	Induces apoptosis; enhances PARP inhibitor effects in TNBC; reduces invasion and metastasis	NCT00296777	Phase Ⅳ	229,231
Escitalopram	Selective serotonin reuptake inhibitor	Induces apoptosis and inhibits non-small-cell lung cancer cell migration	NCT00363909	Phase Ⅲ	233
Sertraline hydrochloride	TCTP inhibitor	Induces apoptosis; promotes autophagy; enhances chemotherapy efficacy	NCT00667121	Phase Ⅲ	318
Solifenacin	Muscarinic antagonist	Alleviates bladder irritation post-TURBT and chemotherapy	NCT01530373	Phase Ⅱ	319
Tanezumab	Anti-NGF antibody	Relieves bone metastasis-related pain	NCT02609828	Phase Ⅲ	238

Note: NaSSA, novel noradrenergic and specific serotonergic antidepressant; TCTP, translation-controlled tumor protein; TCA, tricyclic antidepressant; TURBT, transurethral resection of bladder tumor.

Thioridazine has been reported to suppress tumor growth by inducing G0/G1 phase cell cycle arrest and promoting apoptosis via up-regulation of pro-apoptotic proteins.^215^ Chlorpromazine has demonstrated anti-proliferative effects in breast and colorectal cancer,^221^^,^^222^ while trifluoperazine not only inhibits colorectal cancer cell viability and proliferation but also enhances the efficacy of chemotherapeutics such as 5-fluorouracil and oxaliplatin by inducing apoptosis and repressing CDK activity.^223^^,^^224^ Similarly, fluspirilene has shown synergistic effects when combined with 5-fluorouracil in preclinical studies.^225^ Pimozide reduces cell proliferation, angiogenesis, and metastasis by targeting key pathways such as AKT, VEGF, and MMPs and by inhibiting fibroblast-to-myofibroblast differentiation.^226^ Penfluridol inhibits the integrin α6β4 signaling pathway and has been shown to reduce tumor burden and metastasis in triple-negative breast cancer models.^227^

Beyond antipsychotics, other classes of neuroactive compounds have shown therapeutic promise. The atypical antidepressant mirtazapine, which enhances norepinephrine and 5-HT transmission, alleviates gemcitabine-induced cachexia in pancreatic cancer mouse models and may improve chemotherapy efficacy by modulating the TME.^228^ Tricyclic antidepressants (TCAs) such as imipramine and desipramine, known to influence both serotonergic and noradrenergic systems, have also been implicated in anti-tumor activity.^229^, ^230^, ^231^ Imipramine suppresses breast cancer progression by blocking DNA repair pathways, inducing cell cycle arrest, and enhancing the effects of PARP inhibitors in triple-negative breast cancer, as well as disrupting ER-α signaling in estrogen receptor-positive cancers.^229^^,^^231^ Desipramine enhances cisplatin cytotoxicity by increasing intracellular drug accumulation and activating p53-mediated apoptosis.^230^ Selective 5-HT reuptake inhibitors, such as fluoxetine and escitalopram, primarily used to treat depression and anxiety, have demonstrated anti-cancer effects as well.^232^^,^^233^ Specifically, escitalopram has been shown to inhibit non-small-cell lung cancer cell growth and migration by inducing mitochondria-dependent apoptosis and suppressing NF-κB signaling.^233^

Beyond their potential as monotherapies, several neurotransmitter-targeting agents, such as pimozide, desipramine, and thioridazine, have been shown to enhance the efficacy of conventional chemotherapy and radiotherapy in preclinical models, thereby supporting their integration into combination treatment regimens to further improve therapeutic outcomes.^215^^,^^226^^,^^230^ These findings underscore the potential of incorporating such agents into standard treatment regimens. In particular, combining neurotransmitter-targeting drugs with immunotherapy is an emerging area of interest, as increasing preclinical evidence suggests that neural signaling can modulate the tumor immune microenvironment.^149^^,^^153^^,^^154^^,^^224^ However, clinical trials in this area remain limited, and robust clinical validation is still lacking.

It is important to recognize that the effectiveness of neurotransmitter-targeted therapies may be influenced by cancer type, degree of neural involvement, and TME features. For example, β-blockers have been associated with improved survival in ovarian cancer, whereas their use in lung, breast, or colorectal cancer has not demonstrated a similar benefit.^234^, ^235^, ^236^ Additionally, in pancreatic cancer, the impact of β-blockers remains controversial: one U.S.-based epidemiological study reported no survival benefit, while an analysis using the U.K. primary care database even showed slightly poorer outcomes among users.^235^^,^^237^ These discrepancies underscore the need for precision medicine approaches tailored to tumor context, genetic background, and patient characteristics. Future studies should aim to identify predictive biomarkers of therapeutic response and develop strategies for patient stratification.

Beyond neurotransmitter modulation, targeting neurotrophic signaling pathways also holds significant therapeutic potential. For instance, tanezumab, an NGF inhibitor, may represent a potential strategy to prevent tumor-induced axonogenesis and neurogenesis, as suggested by preclinical findings on the role of NGF in neural remodeling.^75^^,^^76^ Although most current clinical trials with tanezumab have focused on its analgesic effects in cancer-related pain, its potential as an anti-tumor agent targeting the neural niche warrants further investigation.^238^

Another approach involves the disruption of tumor-innervating neural structures. Surgical or pharmacological denervation, using agents such as botulinum toxin or 6-hydroxydopamine (6-OHDA), offers a means to inhibit nerve regeneration and reduce neural support for tumor growth.^125^^,^^214^ Compared with surgical denervation, pharmacological approaches are more controllable and less invasive. Preclinical studies in prostate and gastric cancers have suggested that denervation may delay tumor progression, although off-target effects and lack of specificity remain key challenges.^125^^,^^214^^,^^239^

In addition to promoting tumor progression, nerve–tumor crosstalk plays a central role in cancer-associated pain, particularly in malignancies with rich neural infiltration or PNI. Tumor-derived neurotrophic factors such as NGF and GDNF sensitize peripheral nerves and amplify pain responses.^240^^,^^241^ Preclinical studies and early-phase clinical trials targeting NGF–TrkA signaling (*e.g.*, tanezumab) have demonstrated efficacy in managing metastatic and neuropathic cancer pain.^238^^,^^242^ These findings suggest a dual therapeutic opportunity: targeting nerve–tumor interactions may not only impede tumor progression but also alleviate cancer-associated symptoms. Future clinical trial designs may consider enrolling patients with prominent tumor-related pain, using pain relief as a primary endpoint while concurrently evaluating anti-tumor outcomes.

Despite the compelling biological rationale and supportive preclinical data, clinical translation of nerve-targeting strategies remains limited. Expanding well-designed clinical trials focused on nerve–tumor crosstalk is therefore urgently needed to fully realize the therapeutic potential of this emerging frontier in oncology.

## Emerging technologies for nerve–tumor study

**Figure 6:**
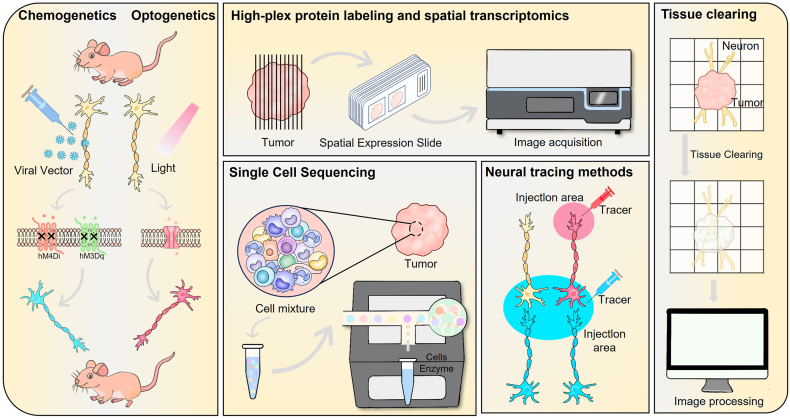
Advances in technologies for nerve–tumor research.

Advancing our understanding of nerve–tumor interactions requires tools capable of capturing cellular complexity, spatial organization, and dynamic signaling. In recent years, technologies such as chemogenetics, optogenetics, single-cell sequencing, and spatial transcriptomics have significantly expanded the toolkit for investigating these interactions (Fig. 6). These approaches not only enable detailed mapping of neural components within the TME but also allow for precise modulation and tracing of neural activity. As these methods continue to evolve, they are poised to uncover previously inaccessible aspects of neurobiology in cancer and offer new opportunities for therapeutic intervention.

### Chemogenetics

Chemogenetics is an innovative technique that integrates genetic engineering and pharmacology to precisely modulate the activity of specific cell populations, particularly neurons.^243^ Central to this approach are Designer Receptors Exclusively Activated by Designer Drugs (DREADDs)-engineered receptors that are selectively activated or inhibited by synthetic ligands, such as clozapine-N-oxide (CNO), without interfering with endogenous signaling pathways.^243^^,^^244^ These receptors enable targeted manipulation of neuronal activity, making them invaluable for studying the nervous system and diseases involving neural dysfunction.

DREADDs are classified based on their functional effects: excitatory DREADDs (*e.g.*, hM3Dq) enhance neuronal firing, while inhibitory DREADDs (*e.g.*, hM4Di) suppress neuronal activity.^245^ These receptors can be introduced into specific neuronal populations via viral vectors, such as adeno-associated viruses (AAVs), or transgenic animal models.^165^ Genetic promoters further ensure cell-type specificity, allowing precise control over neuronal circuits. Ligand administration enables reversible and non-invasive modulation of neural activity, offering unparalleled temporal and spatial precision.

In neuroscience, chemogenetics has transformed our ability to map and manipulate neural circuits, elucidating their roles in behavior, cognition, and disease.^244^^,^^246^ In the context of cancers, chemogenetics provides unique opportunities to study tumor–neuron interactions. By selectively modulating neurons within the TME, researchers can investigate how neural activity influences tumor growth, metastasis, and therapy resistance. Additionally, chemogenetic tools hold the potential for developing targeted therapies, including disrupting nerve-driven tumor progression or enhancing the efficacy of existing treatments.

The high specificity, reversibility, and minimally invasive nature of chemogenetics make it an indispensable tool for advancing our understanding of neuronal function and the role of nerves in cancer. By bridging neuroscience and oncology, chemogenetics opens new avenues for exploring the complex interplay between neurons and tumors, driving innovative research and therapeutic development.

### Optogenetics

Optogenetics is a groundbreaking technique in neuroscience that combines genetic engineering and optical technology to achieve precise control of neuronal activity.^247^, ^248^, ^249^ This approach involves introducing light-sensitive proteins, known as opsins (*e.g.*, channelrhodopsin for activation, halorhodopsin and archaerhodopsin for inhibition), into target neurons.^248^^,^^250^^,^^251^ These opsins enable neurons to be selectively activated or inhibited in response to specific wavelengths of light, allowing researchers to modulate neural circuits in living organisms with millisecond precision.^248^^,^^250^^,^^251^

Compared with chemogenetics, optogenetics offers exceptional temporal resolution, operating on a millisecond timescale.^247^^,^^248^ This makes it uniquely suited for studying dynamic neuronal processes and rapid interactions within neural circuits.^248^ Light delivery can be precisely targeted to specific regions or even individual neurons using fiber optics or advanced imaging systems, enabling unparalleled spatial control.^250^ The flexibility provided by different types of opsins allows researchers to either excite or inhibit neural activity, while the immediate and reversible effects of light stimulation make it ideal for real-time experimentation.^248^^,^^250^^,^^251^

Despite its advantages, optogenetics has notable limitations. The technique requires surgical implantation of fiber optics or light sources for light delivery, which can lead to tissue damage and inflammation.^247^ Targeting deep brain regions is challenging due to light scattering and absorption within the tissue. Additionally, optogenetics demands specialized equipment, such as lasers, optical fibers, and stimulation systems, which can be technically complex and expensive.^247^ Furthermore, prolonged light exposure can cause localized heating, potentially affecting neuronal health and function.^252^

In comparison, while chemogenetics is slower in onset and lacks the temporal precision of optogenetics, it is less invasive, relies on systemic ligand delivery, and does not require sophisticated optical setups. These distinctions highlight the complementary nature of these techniques: optogenetics excels in real-time, high-precision studies, whereas chemogenetics is better suited for long-lasting, systemic investigations. Together, these tools provide researchers with powerful means to dissect the neural circuits involved in nerve–tumor interactions, offering critical insights into how neural activity influences cancer progression and how tumors, in turn, remodel neural components.

### Single-cell sequencing

Single-cell sequencing (SCS) is a transformative tool for studying nerve–tumor interactions, providing unparalleled insights into the cellular and molecular mechanisms underlying this dynamic interplay. It provides a comprehensive view of cellular components in the TME, including cancer cells, immune cells, stroma cells, and neurons.^253^ This ability to resolve cellular heterogeneity and capture dynamic cellular states has made SCS invaluable for understanding the cellular and molecular mechanisms driving nerve–tumor interactions. SCS can detect the expression of neurotrophic factor receptors, such as TrkA and TrkB, on cancer, immune, and stromal cells, and identify their ligands, such as NGF and BDNF, expressed by neurons.^254^, ^255^, ^256^ This enables researchers to uncover receptor–ligand interactions that mediate nerve–tumor crosstalk. Additionally, SCS provides insights into signaling pathways and gene expression changes that facilitate nerve-driven tumor growth, immune modulation, and metastasis, offering a molecular blueprint of this dynamic interaction.

However, SCS has limitations when applied to nerve–tumor interactions. One of its primary drawbacks is the inability to capture the full structure of neurons. Neurons are complex cells with long axons and dendrites that extend far beyond the tumor site, but SCS typically focuses on analyzing single-cell bodies.^257^ This approach excludes critical neural components, such as the nerve fibers actively interacting with tumor cells in the TME. Moreover, neural signals, such as neurotransmitters and neuropeptides, often originate from distant neuronal bodies outside the tumor, which SCS cannot capture. This limitation hinders our understanding of how systemic neural inputs influence tumor behavior.

Another significant limitation is the lack of spatial context. While SCS provides detailed molecular data, it does not preserve the spatial arrangement of nerve fibers and their proximity to cancer or stromal cells within the TME.^258^ This absence of spatial information makes it challenging to study the physical and functional relationships between nerves and tumors or to identify local signaling gradients that drive nerve–tumor interactions. Furthermore, SCS offers only a snapshot of cellular states, lacking the ability to directly reveal functional interactions or causal mechanisms.

### High-plex protein labeling and spatial transcriptomics

The study of nerve–tumor interactions requires advanced technologies that provide spatial, molecular, and cellular insights into the TME. While SCS offers high-resolution molecular profiles, it lacks spatial information.^258^ Emerging technologies such as high-plex protein labeling, 2D spatial transcriptomics, and 3D spatial transcriptomics overcome these limitations by preserving the spatial context of cells within tissues.^258^, ^259^, ^260^

High-plex protein labeling is an advanced technique that allows for the simultaneous detection of multiple proteins within a tissue sample.^259^ Methods such as imaging mass cytometry (IMC), CODEX (CO-Detection by indEXing), and multiplex immunohistochemistry (mIHC) enable researchers to label and visualize dozens to hundreds of protein targets in a single sample.^261^^,^^262^ This technology preserves the tissue architecture, allowing the spatial distribution of nerve fibers, tumor cells, immune cells, and stromal cells within the TME to be identified. Furthermore, high-plex protein labeling provides direct insights into cell signaling dynamics by mapping protein expression and phosphorylation states. Despite its advantages, high-plex protein labeling has limitations. It is restricted to pre-selected protein targets, which may result in the omission of unexpected or novel markers. Additionally, it has a lower throughput compared with transcriptomic approaches, which limits its capacity for large-scale molecular profiling.

2D spatial transcriptomics, such as Visium (10x Genomics) and MERFISH (Multiplexed Error-Robust Fluorescence *In Situ* Hybridization), enable the precise mapping of transcriptomes to their corresponding locations in a 2D plane.^258^^,^^263^^,^^264^ Compared with high-plex protein labeling, 2D spatial transcriptomics offers high-resolution spatial gene expression data and facilitates the discovery of novel spatially regulated genes within the TME.^263^ However, this technique also faces several challenges that may impact its broader applicability. It is restricted to 2D tissue sections, potentially missing critical 3D interactions. Furthermore, it has lower sensitivity than single-cell sequencing, which may hinder its ability to detect rare or low-expressed genes.

Building on the capabilities of 2D spatial transcriptomics, 3D spatial transcriptomics reconstructs gene expression profiles in three dimensions, providing a comprehensive volumetric view of tissues.^260^ Techniques such as STARmap enable high-resolution mapping of gene expression in thick tissue sections while preserving the spatial architecture, thereby facilitating *in situ* 3D transcriptomic analysis.^265^ This holistic view captures complex 3D interactions within the TME, offering unparalleled insights into the spatial relationships between cellular components. However, the high cost and significant technical challenges associated with 3D spatial transcriptomics limit its widespread adoption and routine use in research.

### Neural tracing methods

Neural tracing methods are indispensable tools for mapping the structure and connectivity of neural circuits, offering critical insights into the study of nerve–tumor interactions.^266^ These methods are broadly categorized into anterograde tracing, retrograde tracing, and transsynaptic tracing, with emerging tools further enhancing their precision and applicability.^266^^,^^267^ In the context of nerve–tumor interactions, neural tracing techniques can be employed to identify the origins of nerve fibers infiltrating the TME and to elucidate their connectivity to both the central and peripheral nervous systems. Additionally, these methods are instrumental in highlighting specific neural circuits or pathways that can serve as potential therapeutic targets to disrupt nerve–tumor crosstalk, thereby mitigating tumor growth and spread.

Each tracing technique provides complementary perspectives on neural connectivity: anterograde tracing maps output pathways from neurons, retrograde tracing identifies input connections, and transsynaptic tracing reveals multi-synaptic networks.^268^, ^269^, ^270^ However, to fully understand the physiological impact of nerve–tumor interactions, these structural mapping methods must be integrated with functional approaches, such as optogenetics or electrophysiology. Neural tracing methods, combined with advanced functional techniques, hold significant promise for advancing our understanding of the critical role of nerves in tumor biology and identifying innovative therapeutic strategies.

### Tissue clearing

Tissue clearing renders large tumor specimens optically transparent, enabling light-sheet or confocal 3D visualization of intra-tumoral innervation labeled with neural markers. In nerve–tumor research, clearing supports quantitative readouts: nerve density, branch complexity, and fiber orientation, as well as spatial mapping of PNI and multiplex co-localization with immune and stromal compartments. Common pipelines, such as CLARITY/PACT, iDISCO/iDISCO^+^, CUBIC, and SHIELD, differ in speed, antigen retention, tissue expansion/shrinkage, and compatibility with fresh vs. FFPE samples. The choice of protocol should match epitopes, fluorophores, and desired imaging depth. While powerful, it is constrained by protocol-dependent antigen loss, volumetric distortion requiring scale correction, and depth-related signal attenuation; future refinements should explicitly address these gaps.

In summary, each of these technologies offers unique advantages for studying the interactions between nerves and tumors. However, their respective limitations highlight the need for integration with complementary methods to achieve a comprehensive understanding of the nerve–cancer crosstalk’s molecular and spatial dynamics. For instance, combining spatial transcriptomics with neural tracing can provide a holistic view of both molecular interactions and connectivity, while functional tools such as optogenetics can elucidate the physiological impact of nerve-derived signals on tumor progression. By bridging these technologies, researchers can address current knowledge gaps, identify novel therapeutic targets, and develop innovative strategies to disrupt nerve–tumor interactions, ultimately advancing cancer research and treatment.

## Conclusions

The recognition of nerves as critical players represents the next frontier in cancer research, offering novel insights and therapeutic opportunities. The study of nerve–tumor interactions opens numerous translational and clinical opportunities, from developing innovative therapies to enhancing current treatment modalities.

## CRediT authorship contribution statement

**Liangzhan Sun:** Writing – original draft, Investigation, Conceptualization. **Xia Li:** Investigation, Visualization. **Yaxuan Wang:** Visualization. **Jingxuan Wang:** Investigation. **Renrui Xie:** Investigation. **Ningyi Zhang:** Investigation. **Zemin Zhang:** Supervision.

## Funding

This project was supported by funding from the Chongqing Key Talent Project (China) (No. X9-1358) and Chongqing Medical University Start-up Research Funding (China) (No. J0325001).

## Conflict of interests

The authors disclosed no conflict of interests.
